# Aberrant salience relationship with first rank symptoms

**DOI:** 10.1186/s12991-022-00383-5

**Published:** 2022-02-16

**Authors:** Andrea Ballerini, Marta Tortorelli, Paolo Marino, Cristina Appignanesi, Cinzia Baschirotto, Luca Mallardo, Tommaso Tofani, Francesco Pietrini, Giulio D’Anna, Andrea Rossi, Valdo Ricca, Marina Santella

**Affiliations:** 1 Psychiatry Unit, Department of Health Science, University of Florence, Largo Brambilla 3, 50134 Florence, Italy; 2 Department of Mental Health and Addictions, Central Tuscany NHS Trust, Florence, Italy

**Keywords:** Aberrant salience, Psychotic symptoms, Dimensional approach, Schizophrenic spectrum

## Abstract

**Background:**

Aberrant salience is the incorrect assignment of salience, significance, or value to different innocuous stimuli that might precede the onset of psychotic symptoms. The present study aimed to perform a preliminary evaluation of potentially different correlations between the Aberrant Salience Inventory (ASI) score and dimensional or categorical diagnostic approaches.

**Methods:**

168 adult outpatients with a current psychiatric diagnosis were consecutively enrolled. Patients were evaluated using different psychometric scales. ASI was used to evaluate aberrant salience, and to evaluate the association between ASI scores and first rank symptoms (FRS), and/or with a psychiatric diagnosis. Principal dichotomic clusters of ASI were identified using the Chi-square automatic interaction detection (CHAID) method.

**Results:**

Current (16.76 ± 6.02 vs 13.37 ± 5.76; *p* = 0.001), lifetime (15.74 ± 6.08 vs 13.16 ± 5.74; *p* = 0.005) and past (15.75 ± 6.01 vs 13.33 ± 5.80; *p* = 0.009) FRS were the main clusters dichotomizing ASI. The average ASI score did not significantly differ among patients with different diagnoses.

**Conclusions:**

ASI could be used as a tool to identify psychopathological dimensions, rather than the categorical diagnoses, in the schizophrenic spectrum.

## Keypoints

- Aberrant salience is a trans-nosographic trait of psychotic and pre-psychotic symptoms which is not commonly used in clinical practice.
- The use of the Aberrant Salience Inventory may constitute a valuable instrument for the early detection of psychosis.
- A dimensional approach evaluating aberrant salience may provide a different framework in the assessment of psychotic diagnoses.

## Introduction

The concept of salience was described by Kapur as “a process whereby objects and representations, through the process of association, come to be attention-grabbing and capture thought and behavior” [1]. Several studies suggested the main role of dopamine in this process [2–5] as the mesolimbic dopamine system plays a central role in the attribution of salience [6], converting the representation of a neutral external stimulus into an attractive/aversive one [7]. On the other hand, the dopaminergic hyperactivation in the ventral tegmental area/substantia nigra and striatum is associated with the aberrant feelings of salience [8] and, if chronically maintained, can lead patients through psychotic-like experiences (PLEs) and delusional conclusions [9]. Patients’ PLEs are associated with a plethora of meaningful coincidences impinging on their current worldview, and every single event is perceived as pointing to a new reality. Eventually, delusions become a possible explanation to make sense of these experiences.

According to Kapur [5], patients with aberrant salience over-attribute the meanings of otherwise neutral stimuli, typical in the prodromal stages of psychoses. In fact, at this stage the onset of apprehension and anguish is common for a world that has become uncertain and full of new meanings: This “delusional atmosphere” has been described by Jaspers as “a change which envelops everything with a subtle, pervasive and strangely uncertain light” [10]. This hypothesis might link the aberrant signaling of motivational salience, through the dysregulated dopamine transmission [11], to psychotic symptoms, bridging the gap between neurobiology and phenomenology [1].

Questionnaires providing scores on the proneness to develop psychosis in the general population, including the Magical Ideation Scale [12], the Perceptual Aberration Scale [13], and the Referential Thinking Scale [14], are available. Starting from these scales, Cicero et al. created the Aberrant Salience Inventory (ASI) as a valid and reliable measure of aberrant salience in both the clinical and general population [9]. Even if the interest in this topic is growing, this test is rarely used in research on psychosis, or clinical practice. Furthermore, no study investigates whether aberrant salience is a state or a trait condition and whether aberrant salience assessment can identify proneness to psychosis.

Even if Schneider proposed using the first rank symptoms (FRS) as a reference for the psychiatric diagnostic process in 1959 [15], they are rarely used for clinical assessment; moreover, there is general uncertainty about their diagnostic accuracy [16]. In their review, Soares-Weiser et al. concluded that FRS could be used to help clinicians to assess patients’ clinical conditions, but they do not rely on them alone because FRS appear in the diagnostic criteria of schizophrenia and in other psychotic or non-psychotic mental disorders (FRS are included in the Diagnostic and Statistical Manual—DSM [17] and International Classification of Diseases—ICD [18] checklists). From a psychopathological point of view, psychotic symptoms could be identified in different psychiatric conditions, including affective and psychotic disorders. Using the categorical diagnostic approach, based only on DSM or ICD, the clinician risks misidentifying several psychiatric conditions, especially in their earliest stages, making it practically impossible to identify patients’ aberrant salience experience(s). This misidentification is likely to delay an early therapeutic intervention (psychotherapeutic, psychosocial, or pharmacological) in the prodromal stages of psychosis.

The present study aimed to perform a preliminary evaluation of the potential association between the ASI score with dimensional or categorical diagnostic approaches and, possibly, to identify psychotic proneness in a trans-nosographic real-world population.

## Methods

This is a cross-sectional, monocentric study, conducted by the psychiatric research team at the Adult Psychiatry Unit of Florence University Hospital, according to Good Clinical Practices (GCP), the Declaration of Helsinki (1964), and further revisions. The study protocol was approved by the Ethics Committee of the local institution. Before they were enrolled in the trial, all patients received an information leaflet, in Italian language, explaining exhaustively the protocol and its implications. Further explanations were given by the research team as needed. Before collecting personal data and evaluating clinical conditions, patients were asked to sign their written informed consent.

All outpatients evaluated at the outpatient facility of the Psychiatry Unit of Florence University Hospital, meeting the following inclusion criteria, were consecutively recruited between May and December 2019:

- Age between 18 and 80 years;
- Diagnosis of a psychiatric disorder (depressive disorders, bipolar and related disorders, schizophrenia spectrum and other psychotic disorders, anxiety disorders, obsessive–compulsive and related disorders) according to DSM-V;
- Italian speakers;
- Elementary school degree or above scholarship.

The exclusion criteria were:

- Acute psychotic episode (according to clinical evaluation);
- Cognitive impairment due to psychiatric or medical conditions;
- Diagnosis of a neurological condition.

All diagnoses were assessed by expert psychiatrists (authors AB and VR). Only the main diagnosis was included in the database. The ASI questionnaire was answered by each patient together with a psychiatrist, to ensure the best questionnaire understanding and, thus, the reliability of data collection. One of the research team members was addressed to give any further explanation to patients and to collect socio-demographic and anamnestic data.

The following scales were assessed:

- Functional dimensions of the Manual for the Assessment and Documentation of Psychopathology (AMDP) [19];
- Aberrant Salience Inventory [9];
- Positive and Negative Syndrome Scale (PANSS) [20];
- Montgomery–Asberg Depression Rating Scale (MADRS) [21];
- Hamilton Anxiety Scale (HAMA) [22];
- Mania Rating Scale (MRS) [23];

The presence of Schneider’s FRS was assessed according to the following items in the Manual for the AMDP [24]:

- 33–46 for delusion,
- 47–52 for hallucinations,
- 50–53 for anomalous self-experience.

We have collected the presence of any FRS only, and analyzed it accordingly; AMDP items were not analyzed.

The ASI is a self-reported questionnaire including 29 “Yes”/“No” questions (“Yes”: one point; “No”: zero points), based on the phenomenological descriptions of the prodromal phase of psychosis [9]. According to Kapur’s conceptualization [25], this scale explores five aberrant salience domains:

- Sense sharpening (items 3, 9, 12, 18, and 22);
- Heightened cognition (items 4, 7, 13, 19, 23, and 25);
- Heightened emotionality (items 8, 14, 20, 24, 26, and 28);
- Increased significance (items 1, 5, 10, 15, 16, 21, and 26);
- Impending understanding (items: 2–6–11–17–29).

The Italian version was confirmed to be reliable to identify psychotic patients—who showed higher scores when compared with the general population [26, 27]. Only the total ASI scores were analyzed. An independent research student double-checked the final database, and any discrepancy was aligned with source paper records.

Data were anonymized to guarantee the confidentiality of patients, according to legal requirements, and entered into a computerized database (including personal data, diagnosis, presence of lifetime and current FRS, current pharmacological therapies, questionnaire scores). Only patients who had all ASI items collected were analyzed (per-protocol population).

A preliminary cluster analysis (Chi-square automatic interaction detection, CHAID) was conducted to investigate which of the collected variables dichotomized ASI at a statistically significant level; a step-by-step approach was used. All collected variables (clinical, demographical, psychopathological) were included to determine the first-order cluster discriminative variable. Variables identified as discriminative were excluded from the next steps. A similar analysis was performed on all psychopathological variables to confirm the reliability of the sample and the method. Correlations between total scores of assessed scales were checked.

Continuous variables were compared with Student’s *t*-test; categorical ones were compared with Chi-square.

Statistical significance was set at *p* < 0.05. All statistical analyses were conducted using the default setting of SPSS (Statistical Package for Social Sciences) for Windows 20.0.

## Results

203 consecutive subjects were asked to enter the study, and 7 of them refused to give their consent. Overall, 196 patients were effectively involved in the study, but 28 participants did not fill one or more items of the ASI. 168 subjects completed the entire study protocol, and they were therefore included in our analyses. Socio-demographic and clinical data are summarized in Table 1.

**Table 1:** Socio-demographic and clinical data

	Category	*N*	%
*Sociodemographic variables*			
Gender	Female	95	56.5
	Male	73	43.5
Educational level	Elementary school	7	4.2
	Middle school	39	23.2
	High school	93	55.3
	University	29	17.3
Marital status	Single	66	39.3
	Married	51	30.4
	Divorced	42	25.0
	Widow	9	5.4
Occupation	Student	21	12.5
	Employed	65	38.7
	Unemployed	57	33.9
	Retired	25	14.9
Familial psychiatric anamnesis	Positive	70	41.7
*Clinical variables*			
Diagnosis	Depressive disorders	54	32.1
	Bipolar and related disorders	44	26.2
	Schizophrenia spectrum and other psychotic disorders	54	32.1
	Anxiety disorders	5	3.0
	Obsessive–compulsive and related disorders	11	6.5
FRS	Actual	55	32.7
	Past	80	47.6
	Lifetime	86	51.2

*FRS* first rank symptoms

The age of patients was between 18 and 77 years (mean: 44.38; SD: 15.09). A mood disorder diagnosis was the most common (58.3%). More than half of the patients (51.2%) had experienced at least one FRS during their life. Average HAM-A (12.27 ± 8.12) and MADRS (20.79 ± 12.99) scores highlighted a generalized depressive mood. Furthermore, PANSS scores (average PANSS total 58.08 ± 21.61; PANSS general 33.93 ± 12.53), together with the use of any antipsychotic in 92 subjects (54.2%), confirmed the high frequency of psychotic symptoms in this population (Table 2).

**Table 2:** Psychopathological data

	Mean	Standard deviation
HAM-A	12.27	8.12
PANSS positive	9.79	4.66
PANSS negative	14.52	8.40
PANSS general	33.93	12.53
PANSS total	58.08	21.61
MADRS	20.79	12.99
MRS	4.01	4.10
ASI	14.48	6.04
AMDP		
DAM	2.64	3.68
FTD	5.12	6.96
WeC	3.88	4.16
Delusions	3.85	7.86
DoP	0.65	1.83
EBD	1.63	3.45
DoA	17.89	11.77

*HAM-A* Hamilton Anxiety Scale, *PANSS* Positive and Negative Syndrome Scale, *AMDP* Manual for the Assessment and Documentation of Psychopathology, *MADRS* Montgomery–Asberg Depression Rating Scale, *MRS* Mania Rating Scale, *ASI* Aberrant Salience Inventory, *DAM *Disturbance of Attention and Memory, *FTD* Formal Thought Disorders, *WeC* Worries and Compulsions, *DoP* Disorder of Perception, *EBD* Ego (Boundary Disturbances), *DaA* Disturbances of Affect

The complete comparisons of average ASI values are described in Table 3. Variables dichotomizing the total ASI score are shown in Table 4. Patients with FRS (current, lifetime, or past) had significantly higher ASI scores. When FRS were excluded from the cluster analysis, patients with MRS higher than 3 and PANSS positive symptoms above 7 had significantly higher ASI scores.

**Table 3:** ASI mean scores comparisons by gender and primary diagnoses

Variable	Mean	Standard deviation	*p*
Gender			
Males	14.89	6.29	
Females	14.17	5.86	0.449
FPA			
Positive	14.81	5.65	
Negative	14.24	6.33	0.541
SSPD			
Yes	15.63	6.52	
No	13.94	5.75	0.107
Bipolar			
Yes	14.30	5.61	
No	14.55	6.21	0.803
Depression			
Yes	13.39	5.67	
No	15.00	6.17	0.097
Anxiety			
Yes	12.20	2.95	
No	14.55	6.10	0.153
OCD			
Yes	16.00	7.52	
No	14.38	5.94	0.390

*APS* antipsychotic use, *FRS* first rank symptoms, *FPA* familiar psychiatric anamnesis, *SSPD* Schizophrenia, *OCD* obsessive–compulsive disorder

**Table 4:** ASI score cluster analysis—dichotomizing variables ranked by significance

Discriminant	Mean	Standard deviation	*p*
FRS (actual)			
Yes	16.76	6.02	
No	13.37	5.76	0.001
FRS (lifetime)			
Yes	15.74	6.08	
No	13.16	5.74	0.005
FRS (past)			
Yes	15.75	6.01	
No	13.33	5.80	0.009
MRS			
≤ 3	13.08	5.61	
> 3	16.06	6.15	0.009
PANSS positive			
≤ 7	13.18	5.83	
> 7	15.90	5.97	0.013

*FRS* first rank symptoms, *MRS* Mania Rating Scale, *PANSS* Positive and Negative Syndrome Scale

The cluster analyses also showed that depression diagnosis was the first-step discriminant for MADRS (24.98 ± 10.74 vs 18.80 ± 13.53; *p* = 0.004) and HAMA (14.43 ± 7.51 vs 11.25 ± 8.23; *p* = 0.018), the diagnosis of schizophrenia for PANSS negative symptoms (18.15 ± 9.78 vs 12.78 ± 7.06; *p* < 0.001), while current FRS was the main discriminator for MRS (6.13 ± 4.60 vs 2.98 ± 3.41; *p* < 0.001), PANSS positive symptoms (13.58 ± 6.15 vs 7.95 ± 1.90; *p* < 0.001), PANSS general (38.40 ± 14.12 vs 31.75 ± 11.12; *p* = 0.001) and PANSS total (69.53 ± 24.45 vs 52.51 ± 17.69; *p* = 0.000). No discriminants were found for the other scales collected in this study.

The correlation between scales is summarized in Table 5. While correlation analyses consistently showed a good correlation between scales assessing the same or similar pathological dimensions, none of them showed a significant correlation with ASI.

**Table 5:**
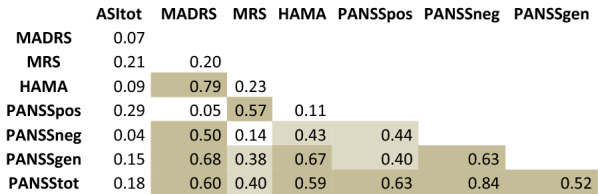
Correlations between scales Correlations (mild > 0.10; medium > 030; high > 0.50)

*HAM-A* Hamilton Anxiety Scale, *PANSS* Positive and Negative Syndrome Scale, *AMDP* Manual for the Assessment and Documentation of Psychopathology, *MADRS* Montgomery–Asberg Depression Rating Scale, *MRS* Mania Rating Scale, *ASI* Aberrant Salience Inventory.

## Discussion

The present study showed that ASI values were higher in patients with FRS, but they did not differ in patients with any specific psychiatric diagnosis. This outcome seemed reasonable, hypothesizing that ASI can vary depending on the current or lifetime presence of psychotic experiences. Furthermore, in the present sample of psychiatric patients, none of the diagnoses seemed to be related to the ASI score. Lelli describes ASI as a scale permitting to discriminate patients from controls, and patients with psychotic symptoms, from patients without them [26], demonstrating its validity and ability to individuate psychotic patients. As in other studies [28], the present study outlined not only the presence of FRS at study assessment but also as past or lifetime, to evaluate whether aberrant salience can be considered mainly a trait feature instead of a state characteristic, independently from the state of illness. Furthermore, the observation of the present confirmed the findings of previous studies, according to which FRS are not specific enough to provide a satisfactory diagnosis [29]. Although these analyses were conducted in a relatively small clinical sample, the results of the correlation analysis confirmed the reliability and consistency of collected data. Thus, the observed outcomes could provide interesting perspectives regarding the use of aberrant salience as a clinically useful paradigm, as part of a psychopathological approach evaluating vulnerability to psychosis recurrence. In this sense, higher values of ASI in patients with current, lifetime, and previous FRS seem to confirm this approach.

As the average ASI score in patients with a main diagnosis of schizophrenia did not significantly differ from the one observed in other psychiatric patients (*p* = 0.107), possibly due to the small sample size, it is possible to hypothesize that salience alteration, underpinned by a dysregulated dopaminergic firing, can be linked with a dimensional and trans-nosographic feature (highlighted by the correlation with FRS), rather than strictly fit into a diagnostic categorization.

According to Howes and Kapur’s III version, “A much more likely scenario is that a biological dysfunction may contribute to one of the major dimensions of the illness. The dopamine dysfunction is present even in subjects reflecting the extended phenotype—family members of people with schizotypya and symptomatic individuals at high risk of psychosis. Thus, the current evidence is consistent with dopamine hyperfunction being most closely linked to the dimension of psychosis […] Dopamine elevation appears specifically related more generally to psychosis proneness and not just to psychosis in schizophrenia” [30]*.*

Considering both Kapur’s hypothesis concerning dopaminergic dysregulation underpinning salience alteration, and Howes’ theorization on altered dopaminergic release as the neurobiological basis of prodromal phases of psychosis [31], we suggest that ASI may be used as a tool to identify prodromal stages of psychosis.

The lack of correlation between ASI and psychiatric scales observed in this study may contribute to confirm its trans-nosographic clinical value. As aberrant salience is described as a part of primary psychotic experiences, this might be a more mindful and reasonable target of a neuroscientific investigation to overcome the established DSM and ICD classifications [32, 33]. The fact that this study was conducted in a single center, where only expert psychiatrists homogeneously performed assessments, potentially reduced the sources of inter-rater variability.

Some limitations of the present study should be acknowledged. First, the present study was performed on a relatively small sample of patients’ afferent to one study center, assessed in a cross-sectional manner, without any follow-up. Second, FRS and psychiatric history were assessed on available anamnestic information, and not by a structured instrument with ad hoc evaluation questionnaires. To conclude, therapies and co-morbidities were not analyzed, and multiple regression models were applicable because of the small sample size.

## Conclusions

The results of this preliminary study seem to confirm ASI validity in identifying psychotic vulnerability. ASI may be used in screening and prevention programs to identify subjects for which a deeper and more careful psychopathological assessment is warranted. Aberrant salience conceptualization [34] may give a different framework in the assessment of psychotic diagnoses, underlining the importance of a dimensional approach in evaluating psychotic patients, through a trans-nosographic perspective. ASI reliability as a predictor of the psychotic disorder needs to be confirmed in a properly designed prospective studies, performed by consistently trained psychiatrists.

## Data Availability

Data are available on request due to privacy/ethical restrictions.

## Abbreviations

AMDP: Manual for the Assessment and Documentation of Psychopathology
ASI: Aberrant Salience Inventory
CHAID: Chi-square automatic interaction detection
DSM: Diagnostic and Statistical Manual
FRS: First rank symptoms
GCP: Good clinical practices
HAMA: Hamilton Anxiety Scale
ICD: International Classification of Diseases
MADRS: Montgomery–Asberg Depression Rating Scale
MRS: Mania Rating Scale
PANSS: Positive and Negative Syndrome Scale
PLEs: Psychotic-like experiences

## References

[1] Kapur S (2003). Psychosis as a state of aberrant salience: a framework linking biology, phenomenology, and pharmacology in schizophrenia. Am J Psychiatry.

[2] Berridge KC, Robinson TE (1998). What is the role of dopamine in reward: hedonic impact, reward learning, or incentive salience?. Brain Res Brain Res Rev.

[3] Berridge KC (2000). Reward learning: reinforcement, incentives, and expectations. Psychol Learn Motiv.

[4] Heinz A (1999). Anhedonia—a general nosology surmounting correlate of a dysfunctional dopaminergic reward system?. Nervenarzt.

[5] Kapur S, Mizrahi R, Li M (2005). From dopamine to salience to psychosis–linking biology, pharmacology and phenomenology of psychosis. Schizophr Res.

[6] Heinz A, Schlagenhauf F (2010). Dopaminergic dysfunction in schizophrenia: salience attribution revisited. Schizophr Bull.

[7] Berridge KC (2012). From prediction error to incentive salience: mesolimbic computation of reward motivation. Eur J Neurosci.

[8] Murray GK, Corlett PR, Clark L, Pessiglione M, Blackwell AD, Honey G (2008). Substantia nigra/ventral tegmental reward prediction error disruption in psychosis. Mol Psychiatry.

[9] Cicero DC, Docherty AR, Becker TM, Martin EA, Kerns JG (2015). Aberrant salience, self-concept clarity, and interview-rated psychotic-like experiences. J Pers Disord.

[10] Jaspers K (1997). General psychopathology.

[11] Berridge KC (2007). The debate over dopamine’s role in reward: the case for incentive salience. Psychopharmacology.

[12] Eckblad M, Chapman LJ (1983). Magical ideation as an indicator of schizotypy. J Consult Clin Psychol.

[13] Chapman LJ, Chapman JP, Raulin ML (1978). Body-image aberration in Schizophrenia. J Abnorm Psychol.

[14] Lenzenweger MF, Bennett ME, Lilenfeld LR (1997). The Referential Thinking Scale as a measure of schizotypy: scale development and initial construct validation. Psychol Assess.

[15] Schneider K (1959). Clinical psychopathology.

[16] Soares-Weiser K, Maayan N, Bergman H, Davenport C, Kirkham AJ, Grabowski S (2015). First rank symptoms for schizophrenia. Cochrane Database Syst Rev.

[17] American Psychiatric Association., American Psychiatric Association. DSM-5 Task Force. Diagnostic and statistical manual of mental disorders: DSM-5. American Psychiatric Association2013.

[18] ICD-10 Version:2010 n.d. https://icd.who.int/browse10/2010/en#!Q77. Accessed 25 May 2019.

[19] Arbeitsgemeinschaft für Methodik und Dokumentation in der Psychiatrie., Scharfetter C. Das AMP-System : Manual zur Dokumentation psychiatrischer Befunde. Springer-Verlag; 1972.

[20] Kay SR, Fiszbein A, Opler LA (1987). The positive and negative syndrome scale (PANSS) for schizophrenia. Schizophr Bull.

[21] Montgomery SA, Asberg M (1979). A new depression scale designed to be sensitive to change. Br J Psychiatry.

[22] Hamilton M (1959). The assessment of anxiety states by rating. Br J Med Psychol.

[23] Young RC, Biggs JT, Ziegler VE, Meyer DA (1978). A rating scale for mania: reliability, validity and sensitivity. Br J Psychiatry.

[24] Arbeitsgemeinschaft für Methodik und Dokumentation in der Psychiatrie, Broome MR, Bottlender R, Rösler M, Stieglitz R-D. The AMDP system : manual for assessment and documentation of psychopathology in psychiatry. n.d.

[25] Jensen J, Kapur S (2009). Salience and psychosis: moving from theory to practise. Psychol Med.

[26] Lelli L, Godini L, Lo Sauro C, Pietrini F, Spadafora M, Talamba GA (2015). Validation of the Italian version of the Aberrant Salience Inventory (ASI): a new measure of psychosis proneness validazione della versione italiana dell’Aberrant Salience Inventory (ASI): una nuova misura per la vulnerabilità alla psicosi. J Psychopathology.

[27] Raballo A, Cicero DC, Kerns JG, Sanna S, Pintus M, Agartz I (2019). Tracking salience in young people: a psychometric field test of the Aberrant Salience Inventory (ASI). Early Interv Psychiatry.

[28] Reininghaus U, Kempton MJ, Valmaggia L, Craig TKJ, Garety P, Onyejiaka A (2016). Stress sensitivity, aberrant salience, and threat anticipation in early psychosis: an experience sampling study. Schizophr Bull.

[29] Nordgaard J, Arnfred SM, Handest P, Parnas J (2008). The diagnostic status of first-rank symptoms. Schizophr Bull.

[30] Howes OD, Kapur S (2009). The dopamine hypothesis of schizophrenia: Version III–the final common pathway. Schizophr Bull.

[31] Howes OD, Montgomery AJ, Asselin M-C, Murray RM, Valli I, Tabraham P (2009). Elevated striatal dopamine function linked to prodromal signs of schizophrenia. Arch Gen Psychiatry.

[32] Maj M (2016). Narrowing the gap between ICD/DSM and RDoC constructs: possible steps and caveats. World Psychiatry.

[33] van Os J (2009). “Salience syndrome” replaces “schizophrenia” in DSM-V and ICD-11: psychiatry’s evidence-based entry into the 21st century?. Acta Psychiatr Scand.

[34] van Os J (2009). A salience dysregulation syndrome. Br J Psychiatry.

